# A Clinical Evaluation of Statin Pleiotropy: Statins Selectively and Dose-Dependently Reduce Vascular Inflammation

**DOI:** 10.1371/journal.pone.0053882

**Published:** 2013-01-22

**Authors:** Evelien van der Meij, Giel G. Koning, Patrick W. Vriens, Marcel F. Peeters, C. Arnoud Meijer, Kim E. Kortekaas, Ronald L. Dalman, J. Hajo van Bockel, Roeland Hanemaaijer, Teake Kooistra, Robert Kleemann, Jan H. N. Lindeman

**Affiliations:** 1 Department of Vascular Surgery, Leiden University Medical Center, Leiden, The Netherlands; 2 Department of Surgery, St. Elisabeth Hospital, Tilburg, The Netherlands; 3 Department of Vascular and Metabolic Diseases, TNO (Toegepast Natuurwetenschappelijk Onderzoek) -Quality of Life, Leiden, The Netherlands; 4 Stanford Medical School, Stanford, California, United States of America; 5 Laboratory for Medical Microbiology, St. Elisabeth Hospital, Tilburg, The Netherlands; Charite Universitätsmedizin, Germany

## Abstract

Statins are thought to reduce vascular inflammation through lipid independent mechanisms. Evaluation of such an effect in atherosclerotic disease is complicated by simultaneous effects on lipid metabolism. Abdominal aortic aneurysms (AAA) are part of the atherosclerotic spectrum of diseases. Unlike atherosclerotic occlusive disease, AAA is *not* lipid driven, thus allowing direct evaluation of putative anti-inflammatory effects. The anti-inflammatory potency of increasing doses (0, 20 or 40 mg/day) simvastatin or atorvastatin was evaluated in 63 patients that were at least 6 weeks on statin therapy and who underwent open AAA repair. A comprehensive analysis using immunohistochemistry, mRNA and protein analyses was applied on aortic wall samples collected during surgery. The effect of statins on AAA growth was analyzed in a separate prospective study in incorporating 142 patients. Both statins equally effectively and dose-dependently reduced aortic wall expression of NFκB regulated mediators (i.e. IL-6 (*P*<0.001) and MCP-1 (*P*<0.001)); shifted macrophage polarization towards a M2 phenotype (*P*<0.0003); selectively reduced macrophage-related markers such as cathepsin K and S (*P*<0.009 and 0.0027 respectively), and ALOX5 (*P*<0.0009), and reduced vascular wall NFκB activity (40 mg/day group, *P*<0.016). No effect was found on other cell types. Evaluation of the clinical efficacy of statins to reduce AAA progression did not indicate an effect of statins on aneurysm growth (*P*<0.337). Hence, in the context of AAA the clinical relevance of statins pleiotropy appears minimal.

## Introduction

Statins are thought to exert anti-inflammatory effects on the vasculature through mechanisms that are independent of their lipid lowering effects [1]. It has been proposed that these so-called pleiotropic effects [2] contribute to the efficacy of these compounds. In vitro and animal studies convincingly show that statins can reduce vascular inflammation by mechanisms that are all unrelated to their effects on the cholesterol metabolism [3], [4]. Yet, it remains to be established whether [5] and how the in-vitro and animal observations translate to the human situation.

An aneurysm of the abdominal aorta (AAA) is a common vascular pathology that is part of the atherosclerotic spectrum of diseases. AAA is a multifaceted pro-inflammatory condition involving macrophages, T-cells, B-cells and neutrophils, as well as multiple pro-inflammatory transcription factors [6], [7]. Unlike atherosclerotic occlusive disease, vascular inflammation in AAA appears not primarily lipid driven. In fact, clear evidence for a role of dyslipidemia in the initiation and progression of AAA is missing [8]. It was thus reasoned that aortic wall samples from AAA patients provide an opportunity to study putative anti-inflammatory effects of statins in a human vascular pathology that is not primarily lipid-driven.

By testing the effect of escalating doses (0, 20 or 40 mg/day) simvastatin or atorvastatin we evaluated the anti-inflammatory potency of regular dose statin to quench molecular as well as cellular facets of vascular inflammation in the AAA. Results of this study show that statins, independent of their lipid lowering effects reduce the expression of selected inflammatory mediators within the vessel wall. These effects are followed by a shift in the M1/M2 balance, and a reduction of markers that are linked to macrophage differentiation. No effect was found on the cellular content or on markers specific for B- and T-cell activation.

## Methods

A total of 63 patients from two centres were included in the study. Sample collection and the coded handling was performed in accordance with the guidelines of the Medical and Ethical Committee of the Leiden University Medical Center, The Netherlands and the code of conduct of the Dutch Federation of Biomedical Scientific Societies (http://www.federa.org/sites/default/files/bijlagen/coreon/codepropersecondaryuseofhumantissue1_0.pdf). Removal of excess aneurysm wall is a standard procedure during open aneurysm repair. As such the vessel wall material in the study is considered “waste” material by Dutch law and as such exempted from ethical consent (WGBO, BW Art. 7∶467) when used in anonymously as was the case in this study.

This is an observational study. As such decisions for statin therapy were based on patient characteristics, local policies, and patient preferences. Decisions were not influenced by the research team. Allocation to the different patients groups was on basis of the statin dose: no statin use (control group, n = 25), intermediate dose (20 mg/day simvastatin or atorvastatin, n = 28) and high dose statin (40 mg/day simvastatin or atorvastatin, n = 10). All statin-treated patients were at least 6 weeks on stable therapy. Plasma total cholesterol, HDL cholesterol and triglyceride levels were measured in a certified clinical chemistry lab. LDL-cholesterol levels were estimated through the Friedewald formula [9].

Decision for open aneurysm repair was based on anatomical (e.g. aneurysm neck, elongation), and patient characteristics (e.g. age) and preferences. Patients with kidney dysfunction (estimated clearance <30 mL/min), chronic inflammatory disease or (suspected) so-called inflammatory aortic aneurysms were excluded from participation in the study.

Aortic wall tissue was taken from the anterior-lateral aneurysm wall at the level of the maximal diameter of the aneurysm. Samples were collected immediately after opening of the aneurysm sac. Adhering thrombus was carefully removed and wall samples were immediately halved. One half was snap-frozen in CO_2_-cooled iso-pentane or liquid nitrogen, and stored at −80°C until use for mRNA (RT-PCR) and protein (multiplex assay, ELISAs, Western blots and protease activity assays) analysis. The other half was fixed in formaldehyde (24 h), decalcified (Kristensens solution, 120 h), and paraffin-embedded for histological analysis [7].

All analyses were performed in an investigator-blind fashion.

The effect of statin therapy on aneurysm growth was evaluated in the control (placebo) cohort of the Pharmaceutical Aneurysm Stabilization Trial (PHAST) (NTR 1345). PHAST is a placebo controlled multi center trial of 18 months doxycycline vs. placebo in patients under surveillance for a small AAA (35–50 mm) with aneurysm progression as the primary endpoint. Primary approval for the study was granted by the Medical and Ethical Committee of the Leiden University Medical Center, The Netherlands. All local review boards of the participating centers approved the study. Written informed consent was obtained from all participants. The PHAST study is supported by grants from ZonMw (The Netherlands Organisation for Health Research and Development) grant 40-41200-98-052 and NutsOhra Fund (project 0701-061). The funding sources were not involved in the process of the study or manuscript preparation

The placebo group consisted of 142 patients, 103 of them were on statin therapy, and 39 were not on a statin. AAA progression was measured by standardized ultrasound at 6-month intervals. All measurements were made by a single observer. Decisions for statin therapy were based on patient characteristics, local policies, and patient preferences and were not influenced by participation the study.

### Immunohistochemistry

Immunohistochemistry was performed on deparaffinized, ethanol rehydrated 4 µm cross-sections and using established antibodies. Cross-sections were incubated overnight with antibodies specifically staining human myeloperoxidase (rabbit polyclonal, 1∶4000 dilution, DakoCytomation, Heverlee, Belgium), CD3 (polyclonal rabbit, 1∶400 dilution, Abcam, Cambridge, UK), CD4 (clone 4B12, 1∶200 dilution, DakoCytomation), CD8 (clone C8/144B, 1∶200 dilution, DakoCytomation), CD20 (clone L26, 1∶1000 dilution, DakoCytomation), CD68 (clone KP6, 1∶1200, DakoCytomation), CD138 (clone B-B4, 1∶1000 dilution, Serotec, Oxford, UK) [7].

Macrophage differentiation (M1/M2) and activation was estimated by double staining (control group and 40 mg simvastatin/atorvastatin only). CD68 (clone L26, DakoCytomation)/iNOS (rabbit polyclonal AB3523, 1∶400 dilution, Abcam Cambridge, UK) double staining for M1 macrophages, and CD68/CD163 (Clone 10D6, 1∶400 dilution, DakoCytomation) for M2 macrophages. Double staining for CD68/HLA-Dr (clone TAL.1B5, 1∶1000 dilution, DakoCytomation) was performed in order to evaluate a possible effect on macrophage activation. Sections were quantified by counting the number of double positive cells in 6 representative medium power fields (2 photographs for each of the vascular layers). Sections were quantified in a blinded fashion by two independent observers. Since AAAs are characterized by a transmural inflammation the total number of cells counted was used in the analysis.

### RNA extraction, mRNA analysis and NFκB DNA binding

Total RNA extraction was performed using RNAzol (Campro Scientific, Veenendaal, The Netherlands) and glass beads [10]. Complementary-DNA was prepared using kit #A3500 (Promega, Leiden, The Netherlands) and quantitative real-time polymerase chain reaction (QRT-PCR) analysis was performed for human IL-1α, IL-1β, IL-6, IL-8, TNFα, MCP-1, TGFβ, COX2, ALOX5, GATA-3, T-Bet, IL-4, IL-10, IL-13, Perforin, Granzyme A, BLIMP-1, MAD4, Immunoglobulin linkerprotein, Immunoglobulin heavy chain, Tissue Factor, MMP-25, MMP-8, MMP-9, MMP-12, MMP-13, TIMP-1, Cathepsin K, Cathepsin L, Cathepsin S, on the ABI-7500 Fast system (Applied Biosytems, Nieuwerkerk a/d IJssel, The Netherlands) using established primer/probe sets (Assays on Demand, Applied Biosystems) and Taqman Gene Expression Master Mix (Applied Biosystems). Analyses were performed according to the manufacturer's instructions and as previously reported [11]. Relative expression was calculated using the delta delta CT method. Glyceraldehyde-3-phosphate dehydrogenase (GAPDH) expression was used as a reference and for normalization.

NFκB DNA binding activity was assessed by the TransAM™ Chemi Kit NFκB DNA-binding ELISA (Active Motive, Rixensart, Belgium) as reported previously [12]. To that end nuclear extracts were prepared from the homogenates (Active Motive nuclear extract kit), and the amount of active p65-NFκB DNA was assessed in the extracts.

### Tissue homogenization and protein analysis

Aortic wall tissues were pulverized in liquid nitrogen and homogenized in 2 volumes lysis buffer (10 mM Tris pH 7.0, 0.1 mM CaCl_2_, 0.1 M NaCl, 0.25% (^v^/_v_) Triton X-100). This method releases both soluble and membrane bound proteins. Samples were subsequently centrifuged at 10,000 g for 15 minutes at 4°C, and the supernatant protein extract was snap-frozen in liquid nitrogen and stored at −80°C until use. Protein content in thawed protein extracts homogenates was determined with a BCA protein assay kit (Pierce, Rockford, IL, USA).

Cytokine/chemokine protein levels in the homogenates were measured by the bio-plex panel for multiple cytokines (Bio-Rad Laboratories B.V, Veenendaal, The Netherlands; IL-1α, IL-1β, IL-13, TNFα, IFN-γ, IP-10, MIP-1α, MIP-1β and G-CSF) or by separate ELISAs (PeliKane compact kit (Sanquin, Amsterdam, The Netherlands) for IL-6 and IL-8 and the Quantikine kit (R&D Systems, Abingdon, UK) for MCP-1. We previously showed that IL-2, IL-4, IL-5, IL-7, IL-10, IL-12, IL-17A and GM-CSF levels in AAA are all at or below the detection threshold of the multiplex assay [7]. Hence, these cytokines were not included in the analysis.

Soluble ICAM levels were measured in a Quantikine ELISA from R&D systems (Abingdon UK).

Quantifiable Western blots were performed following detailed protocols described by Kleemann and colleagues [13] using the following antibodies: Cathepsin K (IM55L, Calbiochem, Breda, The Netherlands); Cathepsin S (sc-6505, Santa Cruz) and α-actin (sc-1615, Santa Cruz) for normalization. All secondary antibodies were obtained from Abcam (Cambridge UK). Immunoblots were visualized using Super Signal West Dura Extended Duration Substrate (Perbio Science, Etten-Leur, the Netherlands), and a luminescent image workstation (UVP, Cambridge, UK). Immunoblots were quantified using LabWorks 4.6 software.

MMP8 and 9 activity assays (Amersham Biosciences, Buckinghamshire, UK) were performed according to the supplier's recommendations [12].

### Statistical analysis

Sample size estimates were based on the assumption that the effects exerted by statins on aortic wall IL-6 levels were equal to the effects observed after doxycycline therapy in an earlier study [14]. On basis this data from a group size of 25 patients would allow a power of 90% with an alpha of 0.05 (two-sided).

Statistical analyses were performed with SPSS16.0. No difference was found between simvastatin or atorvastatin for all markers tested. Therefore, data for the two statins were combined. Differences between the groups were evaluated by One-Way ANOVA (general linear model) or the Kruskal Wallis test in case of non-normal distributed continuous data. Fisher's exact test was used for the evaluation of categorical data. All normally distributed values are expressed as means (SD). Medians [inter quartile range (IQR)] are provided in case of non-normally distributed data. Simple regression analysis and multiple regression analysis was performed to evaluate the relationships between aortic wall MCP-1, IL-6 content, and plasma cholesterol levels.

This paper contains multiple comparisons between largely coherent data that fit in theoretical inflammatory frame works. Application of very stringent correction methods for multiple comparisons such as the Bonferroni methodology may lead to unacceptable large type II errors. For this reason we decided for the Benjamini and Hochberg False Discovery Rate correction (family wise) to dictate the level of significance.

We used linear mixed-effects models to examine a possible effect of statin therapy on changes in aneurysm diameter during the follow-up period [15]. Random terms in the models were patient, follow-up time and the square of follow-up time, with an unstructured covariance matrix. Fixed terms were follow up, follow up squared and the interactions of the latter two with treatment group. Baseline diameter was used as dependent variable and the model did not allow a systematic difference at baseline between the treatment groups. The effect of statin treatment on aneurysm growth at 18 months follow up was estimated and tested based on this model, as was the difference at 6 and 12 months. Models were validated both graphically by residuals analysis and analytically by extending the models with more terms. Baseline characteristics in the PHAST study were compared by unpaired t-tests in the case of numerical variables. The chi square test was used for categorical data.

## Results

### Patients

A total of sixty-three patients were enrolled in the study. Patients were divided on basis of their statin use in a control group, not using a statin (n = 25); a group using intermediate dose (20 mg/day) simvastatin or atorvastatin (n = 28); and a group using standard dose (40 mg/day) simvastatin or atorvastatin (n = 10). Patient characteristics are shown in Table 1. No statistical differences were found with respect to sex, age, smoking status and AAA diameter. Total and LDL cholesterol levels in the 20 mg group (median levels: 4.10 and 2.33 mmol/L) were lower than those in the control group (4.97 and 2.88 mmol/L, P<0.004 and P<0.009 respectively). Total cholesterol levels in the 40 mg simvastatin/atorvastatin group (4.76 mmol/L) were similar to the levels in the 20 mg simvastatin/atorvastatin group but the LDL cholesterol levels (3.39 mmol/L) were marginally lower (P<0.05).

**Table 1:** Patient characteristics mean (sd) or median [25^th^–75^th^ percentile].

		Simvastatin/Atorvastatin		
	No Medication	20 mg	40 mg	*P*
Evaluable patients	25	28	10	
Mean Age (yr, median, IQR))	70 [68–80]	69 [64–72]	68 [65–72]	0.08
AAA diameter (cm, mean ± sd)	6.3 (1.2)	6.1 (1.0)	5.5 (0.9)	0.12
Female sex	2	7	4	0.08
Smoking	1/13/4/7	4/11/5/8	1/7/0/2	0.60
(Unknown/Current/Never/Former)				
ACE Inhibitor	7	7	4	0.70
Total Cholesterol (mmol/L)	4.97 [4.15–6.02]	4.10 [3.62–4.55]	4.76 [3.81–5.58]	1)
LDL-Cholesterol (mmol/L)	2.88 [2.76–3.54]	2.33 [1.85–2.76]	3.39 [2.50–3.85]	2)
HDL Cholesterol (mmol/L)	1.24 [0.85–1.58]	1.03 [0.92–1.54]	1.11 [1.03–1.28]	0.83
Triglycerides (mmol/L)	1.31[1.03–1.80]	1.34 [0.92–1.74]	1.16 [0.83–1.57]	0.67

1) P is <0.004 and P<0.009 respectively for no statin vs 20 mg simvastatin or atorvastatin and 20 mg vs 40 mg simvastatin or atorvastatin;

2) P = ns for no statin vs 20 mg simvastatin or atorvastatin and P<0.05 for 20 mg vs 40 mg simvastatin or atorvastatin.

The effect of statin therapy on aneurysm progression was studied in the control arm of the PHAST-study. Patient characteristics of this cohort are shown in Table S1. 103 of the 142 patients were on statins; with the exception of a history of cardiovascular disease, antihypertensive use and plasma LDL levels groups were well balanced.

Statin therapy and the cellular response in AAAAAAs are characterized by a broad cellular inflammatory component consisting of macrophages, neutrophils, and T- and B-cells [6], [7]. Immuno-histochemical assessment of the relative abundance of the different inflammatory cell types (i.e. monocytes/macrophages (CD68^+^), neutrophils (MPO^+^), T-cells (CD3^+^), T-helper cells (CD4^+^), cytotoxic T-cells (CD8^+^), B-cells (CD20^+^) and plasma cells (CD138^+^)) did not indicate an effect of statin therapy on cellular content (Figure S1) or distribution (not shown).

### Statin therapy and aortic wall cytokine/chemokine levels

**Figure 1:**
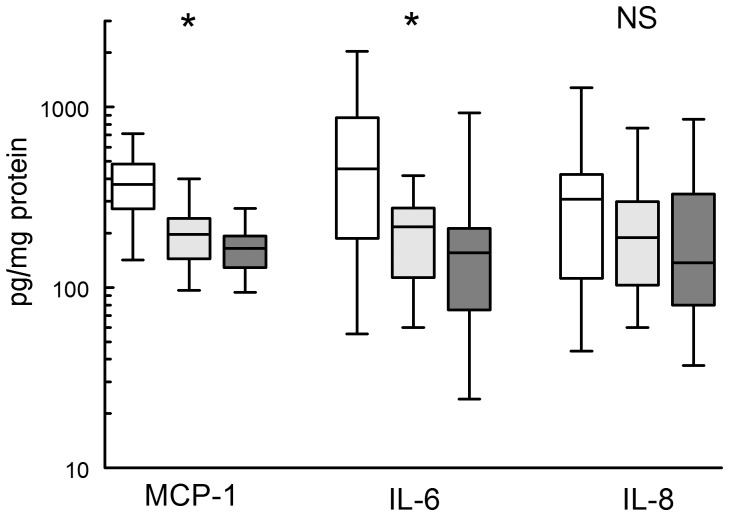
The effect of statin treatment on aortic wall MCP-1, Il-6 and IL-8 levels. Non-treated controls (white bars); 20 mg statin treated (grey bars) and 40 mg statin treated (black bars). ^*)^ significant and dose dependent reduction, P<0.0003. NS: not significant.

**Figure 2:**
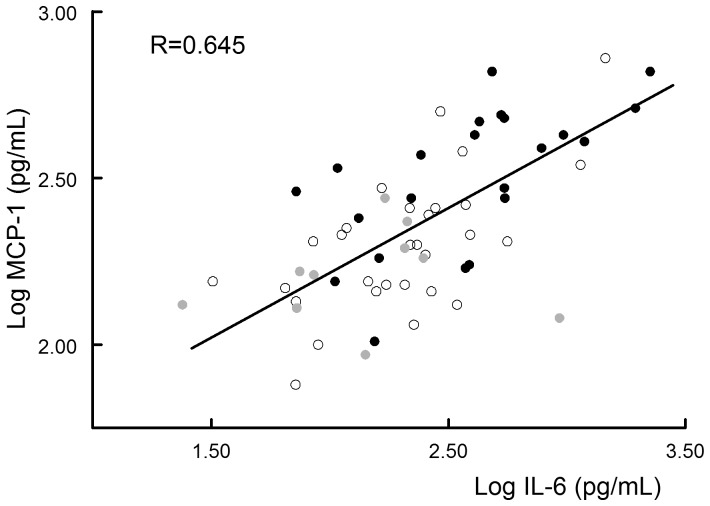
There is a strong correlation between aortic wall IL-6 and MCP-1 levels, p<0.0001. Black circles: controls; Open circles: Simvastatin/Atorvastatin 20 mg, Grey circles: Simvastatin/Atorvastatin 20 mg.

Evaluation of the effect of statin treatment on inflammatory mediators showed a clear dose-dependent reduction of aortic wall IL-1, IL-6, TNFα and interferon γ mRNA levels (Table 2). At the protein level, statins dose-dependently reduced IL-6 and MCP-1 levels from median levels of 455 (IL-6) and 344 (MCP-1) pg/mg protein in the control group to 156 and 164 pg/mg protein, respectively in the 40 mg simvastatin/atorvastatin group (Figure 1, *P*<0.0073 and P<0.00014 respectively). The decreased MCP-1 levels were accompanied by reduced levels of the MCP-1 ligand ICAM-1 (*P*<0.0018, table 3). No effects were found on other cytokines and chemokines such as IL-8 (*P*<0.17, Figure 1) and the interferon γ inducible protein 10 (IP-10) (Table 3). With the exception of a moderate relationship between aortic wall IL-6 levels and plasma cholesterol (R = 0.39, *P*<0.008), no relationship was found between plasma cholesterol and any of the cytokines or chemokines. A remarkably strong correlation was observed between aortic wall IL-6 and MCP-1 protein levels (R = 0.65, *P*<0.0001, Figure 2). Stepwise regression analysis suggested that these relationships between IL-6, plasma cholesterol and MCP-1 reflect distinct mechanisms (R for the model incorporating plasma cholesterol and MCP-1: 0.79; β_plasma cholesterol_ 0.27 (*P*<0.008); β_mcp-1_ 0.69 (*P*<0.0001)).

**Table 2:** Relative mRNA expression (log transcript level relative to GAPDH (GAPDH = 0)) of inflammatory mediators, cell activation markers and proteases and the MMP inhibitor TIMP-1.

		Controls	Simvastatin/Atorvastatin		
		N = 22	N = 28	N = 10	*P*
Cytokines	IL-1α	−1.46 [−1.81, −1.27]	−1.71 [−2.19, −1.28]	−2.14 [−3.06, −0.83]	0.003*)
	IL-1ß	−0.26 (0.40)	−0.54 (0.55)	−1.05 (1.14)	0.002*)
	IL-6	−0.12 (0.51)	−0.31 (0.51)	−0.66 (1.15)	0.038*)
	IL-8	−0.61 (0.53	−0.70 (0.50)	−0.97 (0.63)	0.098
	TNFα	−1.16 [−1.10, −1.82]	−1.32 [−1.82, −1.10]	−1.75 [−2.47, −1.46]	0.014*)
	MCP-1	−0.23 (0.46)	−0.34 (0.40)	−0.52 (0.95)	0.256
	Interferon γ	−1.90 (0.43)	−1.80 (0.60)	−2.61 (0.74)	0.021*)
	TGFß	−0.75 [−1.01, −0.61]	−0.83 [−1.09, −0.63]	−0.60 [−0.91, 0.11]	0.542
T-helper cell	GATA-3	−1.63 (0.41)	−1.53 (0.40)	−2.02 (0.79)	0.113
	T-Bet	−1.77 (0.33)	−1.72 (0.36)	−2.30 (0.75)	0.020*)
	IL-4	−2.16 [−3.35, −1.87]	−2.32 [−2.69, −1.85]	−3.52 [ND, −2.07]	0.315
	IL-10	−1.00 [−1.47, −0.77]	−0.83 [−1.34, −0.67]	−1.33 [−2.39, −0.86]	0.637
	IL-13	−1.87 [−2.87, −1.49]	−2.63 [ND, −1.80]	ND [−4.11, −1.94]	0.011*)
Cytotoxic T cell	Perforin	−2.04 [−2.35, −1.68]	−1.98 [−2.26, −1.71]	−2.35 [ND, −1.59]	0.406
	Granzyme A	−0.56 (0.32)	−0.47 (0.79)	−1.20 (1.22)	0.095
B/Plasma cell	MAD4	−0.81 (0.18)	−0.84 (0.38)	−1.00 (0.83)	0.316
	BLIMP	0.98 [0.70, 1.44]	0.98 [0.48, 1.21]	0.79 [−0.780, 1.29]	0.319
	IgG heavychain	1.18 (0.54)	1.27 (0.83)	0.68 (1.71)	0.207
	IgG linker protein	1.11 (0.61)	1.28 (1.14)	0.44 (1.17)	0.179
Neutrophil	MMP-25	−2.85 [ND, −1.85]	−2.00 [ND, −1.70]	−2.26 [ND, −1.46]	0.307
Macrophage	MMP-12	0.08 (0.25)	0.17 (0.22)	−0.05 (0.71)	0.583
	Cathepsin K	0.93 (0.43)	0.64 (0.96)	−0.12 (1.66)	0.009*)
	Tissue factor	−0.81 (0.37)	−0.67 (0.42)	−1.17 (0.88)	0.031*)
	COX-2	0.46 (0.28)	1.00 (0.66)	0.51 (1.37)	0.165
	ALOX-5	−0.32 (0.36)	−0.30 (0.30)	−0.89 (0.75)	0.0009*)
	iNOS	−2.20 [−2.93, −0.99]	−1.38 [−1.94, −0.86]	−1.73 [−3.13, −1.57]	0.748
Proteases	MMP-8	−2.46 [ND, −2.14]	ND [ND, −2.39]	−3.07 [ND, −1.85]	0.659
	MMP-9	−0.66 (0.34)	−0.84 (0.80)	−0.98 (1.11)	0.222
	MMP-13	−1.15 (0.54)	−1.18 (0.80)	−1.62 (1.19)	0.128
	TIMP-1	−1.43 [ND, −1.43]	−1.19 [−1.83, −0.75]	−1.16 [−3.30, −0.69]	0.156
	Cathepsin L	−0.19 (0.43)	−0.15 (0.31)	−0.48 (0.71)	0.166
	Cathepsin S	0.46 (0.28)	0.27 (0.64)	−0.09 (1.06)	0.027*)

Mean (sd) or median [IQR].

* P value is for a linear trend between the groups in case of normally distributed data (general linear model in one-way ANOVA), or for the Kruskal Wallis test in case of non-normal distributed data.

*) significance reached after the Benjamini and Hochberg False Discovery Rate correction.

**Table 3:** Effect of statin treatment on aneurysm wall cytokine and chemokine protein expression ((pg/mg protein), median [IQR], assessed by the BioRad panel for multiple cytokines).

	Controls	Simvastatin/Atorvastatin		
	N = 22	N = 29	N = 10	
IL-1α	0.14 [ND – 0.34]	ND [ND – 0.11]	ND	0.106
IL-1ß	2.81 [2.0–7.54]	3.18 [1.42–5.41]	1.50 [0.91–2.70]	0.045
IL-13	0.18 [0.90–0.28]	0.03 [ND –0.09]	0.01 [ND –0.07]	0.005*)
TNFα	0.04 [0.01–0.11]	0.03 [0.01–0.09]	0.03 [ND –0.07]	0.548
Interferon γ	1.32 [ND –3.62]	0.13 [ND –2.93]	0.85 [ND –1.74]	0.321
IP-10	141.2 [63.0–237.0]	99.2 [40.7–184.4]	63.6 [34.4–109.6]	0.182
MIP-1α	4.65 [3.52–9.81]	3.80 [1.95–5.17]	2.01 [0.80–5.64]	0.269
MIP-1β	20.01 [10.10–26.56]	7.49 [2.61–17.16]	2.73 [1.18–17.52]	0.369
G-CSF	0.73 [0.40–1.77]	0.49 [ND–1.25]	0.25 [ND–0.76]	0.051
Log sICAM	4.04 (0.27)	4.10 (0.32)	3.66 (0.43)	0.0018*)

IL-4, IL-5, IL-10, IL-12 and IL-17A levels were all below the detection threshold of the assay and are therefore not shown in the table. Levels of IL-6, IL-8 and MCP-1 exceeded the upper detection threshold and were, along with sICAM-1 accurately quantified in separate ELISA's. ND: not detectable: below the detection threshold of the assay. *P* value is for the Kruskal Wallis test for non-normally distributed data.

*) significance reached after the Benjamini and Hochberg False Discovery Rate correction.

### Effects of statin therapy on aortic wall leukocyte activation

We next tested whether the observed differences in the inflammatory mediators are associated with a change of the cellular activation status. As shown in tables 2 and 3 statin therapy had a modest effect on the T-Bet and IL-13 expression, but other than that it did not influence the polarization or activation status of T-helper cells (assessed by mRNA expression of the activation/polarization markers GATA-3, IL-4, and IL-10) or the activation status of cytotoxic T-cells (assessed by perforin and granzyme A), B cells/plasma cells (BLIMP-1, MAD-4, Ig linker protein, IgG heavy chain) or neutrophils (MMP-25 mRNA expression, and MMP-8 and MMP-9 proenzyme levels (results no shown)).

**Figure 3:**
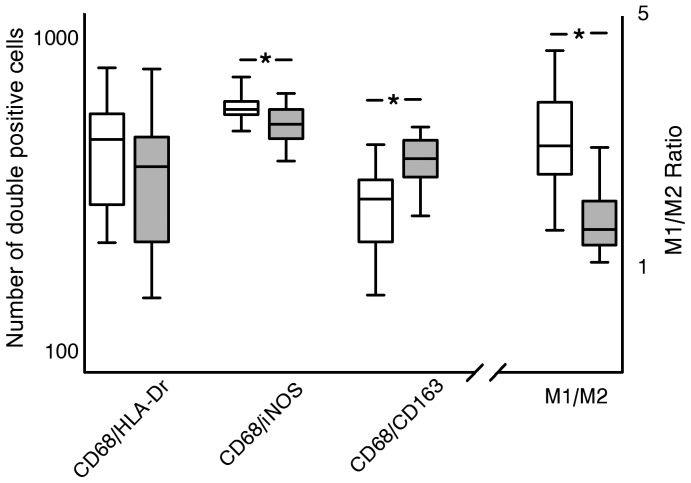
Statin therapy does not influence macrophage activation (number of CD68/HLA-Dr positive cells, but reduces aortic wall M1 (CD68/iNOS double positive cells, P<0.019) and increases M2 (CD68/CD163 double positive cells, P<0.001) content, thereby resulting in a shift in the M1/M2 balance (P<0.0003). Cell counts reflect the number of double positive cells per 6 medium power fields. Non-treated controls (white bars); 40 mg statin treated (grey bars).

**Figure 4:**
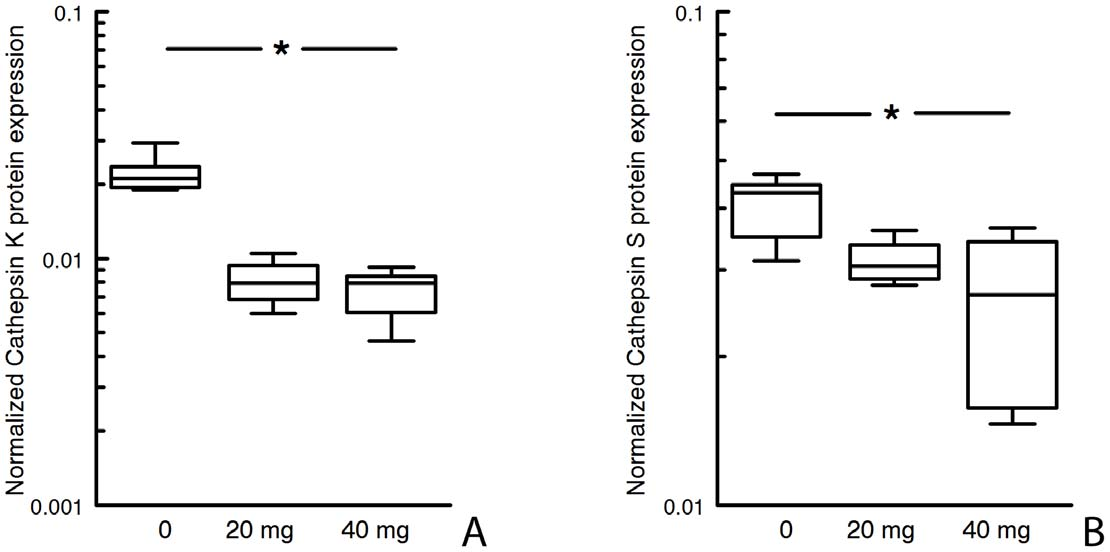
Relative aortic wall cathepsin K and S expression assessed by Western blot analysis (normalized on basis of actin levels). *Levels significantly reduced compared to non-treated controls, P<0.034.

Statin therapy was associated with a clear reduction in the number of M1 (p<0.019) and an increase in the number of M2 macrophages (*P*<0.001), thus resulting in a clear shift in the M1/M2 balance (p<0.0003) towards more alternatively activated phenotype (M2) (Figure 3, Figure S2). Double staining for CD68/HLA-Dr did not indicate an effect on macrophage activation level (Figure 3). Mixed effects were observed for markers that have been linked to macrophage differentiation. No effect was found for iNOS, COX-2, and MMP9 and -12 (macrophage elastase) expression, but we observed a dose-dependent reductions of arachidonate lipoxygenase-5 (ALOX5) (*P*<0.0009), and of cathepsin K and S mRNA (*P*<0.009 and 0.0027) and protein expression (*P*<0.011 and *P*<0.034 respectively; Table 2; Figure 4, Figure S3).

### Effects of statin therapy on NFκB activation

**Figure 5:**
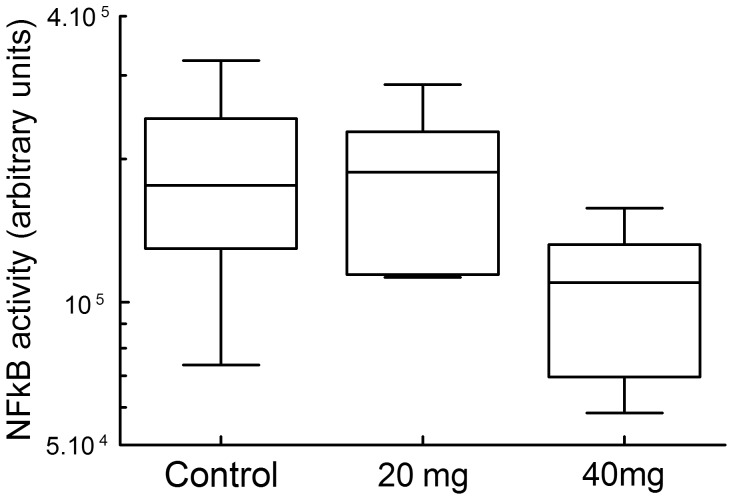
Aneurysm wall NFκB activity (active p65-NFκB DNA binding assessed in the TransAM™Chemi-Kit NFκB DNA-binding ELISA). *NFκB activity significantly reduced compared to the control group (P<0.016).

The effect of statin therapy on primarily NFκB regulated inflammatory mediators (IL-1α and β, IL6, TNFα, interferon γ and MCP-1), and the strong co-variation between the NFκB regulated genes MCP-1 and IL-6 suggests that statins may act through a selective effect on aortic wall NFκB activation. This notion is supported by a 40% reduction in NFκB DNA binding in the 40 mg/day statin group, (Figure 5, *P*<0.016) and by reduced Tissue Factor expression, a classical NFκB regulated gene in statin treated patients (*P*<0.031, Table 2).

### Statin therapy and aneurysm growth

**Figure 6:**
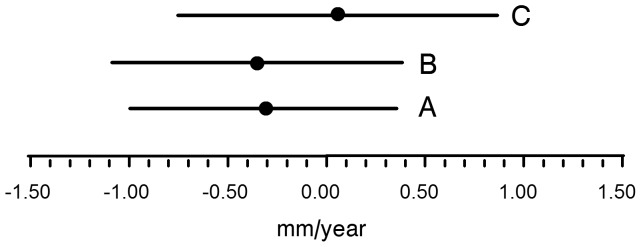
The effect of statin therapy on aneurysm progression (mean, 95% confidentiality interval in mm/year). A: corrected for base line diameter, age and gender (P<0.36); B is correction A plus correction for diabetes mellitus, history cardiovascular disease, anti hypertensives, aspirin and smoking (P<0.34); and C is correction B plus correction for cholesterol (P<0.89).

A critical point is whether and how the above effects influence the disease process. Currently the effect of statins on aneurysm growth is a controversial and disputed subject. In this context we considered an evaluation of the effects of statin therapy on aneurysm progression relevant. To that end we analyzed the effect of statin therapy on aneurysm progression in the control group of the PHAST study. Clinical characteristics of the two subgroups are shown in Table S1. Results from this analysis show equal aneurysm progression in those with and those without statin treatment (*P*<0.36 after correction for baseline diameter, gender and and age (model A) and *P*<0.90 for a more comprehensive correction (Figure 6)).

## Discussion

We here show that statin se is associated with a dose-dependent reduction of the expression of selected inflammatory mediators by a mechanism involving reduced vascular NFκB activation in human AAA, a vascular pathology that is not primarily lipid driven [8]. Quenching of inflammatory intermediates in the vessel wall was paralleled by a selective reduction of markers that are linked to macrophage differentiation.. Despite clear reductions in pro-inflammatory cytokines, and cysteine protease levels, statin treatment failed to reduce aneurysm progression.

Sub-analyses show a similar efficacy of simvastatin and atorvastatin. This observation follows clinical observations showing an equal effectiveness of simvastatin, and atorvastatin in long-term cardiovascular prevention [16].

Statins are highly efficient cholesterol lowering drugs that act through inhibition of the enzyme HMG-CoA reductase. Observed reductions in circulating inflammatory markers (e.g. CRP) during statin therapy led to the view that statins, independent of their lipid lowering effects, may also influence the inflammatory cascades. It was thus proposed that the clinically beneficial effects of statin therapy are partially related to an anti-inflammatory effect on the vasculature (so called pleiotropic effect) [1], [2]. Despite clear in vitro and animal evidence for off-target pleiotrope effects on vascular inflammation [3], [4]. there is little, if any evidence for a pleiotropic effect of statin therapy in the cumulative data of the major cholesterol lowering trials [5], [17]. Conversely, clinical observations such as the efficacy of statins in preventing cerebrovascular disease (a manifestation of atherosclerotic disease that is not clearly associated with hypercholesterolemia) [18], and the almost instantaneous beneficial action of statin therapy in patients with unstable angina (an effect attributed to plaque stabilization) [19] favour an effect beyond that of simply lipid lowering.

Direct evaluation of putative pleiotropic effects and the interpretation of the findings in human studies is hindered by inherent simultaneous effects of statin therapy on the lipid profile, which may result in secondary reductions in vascular inflammation. Another potential interfering factor is that the majority of statin trials have been performed with doses that are currently considered low or intermediate. Since the threshold for the anti inflammatory effects exceeds that of the lipid lowering [20], potential pleiotropic effects may be missed when extrapolating data from the large scale statin trials (as in reference [18]).

Considering the long standing debate on the pleiotropic effects of statins on vascular inflammation, and the inherent problems when attempting to separate direct effects from indirect effects (i.e. changes brought about by a concomitant effect on the lipid profile) in human atherosclerotic disease, we set out to explore the effects of statin therapy on vascular inflammation in the abdominal aneurysm. Although this is pathology is considered part of the atherosclerotic spectrum of diseases [21] there is little evidence for a role of dyslipidemia in the initiation and/or progression of AAA [8]. This notion is supported by the absence of a relationship between plasma cholesterol levels and aneurysm wall inflammatory parameters in this study.

Moreover, we previously characterized AAA as a broad inflammatory condition involving multiple proinflammatory pathways and inflammatory intermediates [7]. Hence, the pathology allows for simultaneous evaluation of a multiplicity of inflammatory pathways. The feasibility of such an approach is shown in our studies evaluating the effects of doxycycline treatment on vascular wall proteases and inflammation in AAA [^1^2]. Results of the present study show that statins have a selective, dose dependent effect on, predominately NFκB regulated inflammatory mediators; suggesting that statins specifically quench NFκB activity mediated processes. We assessed aortic wall p65-NFκB activity in a highly specific assay, and found an approximate 40% reduction in p65-NFκB activation in the 40 mg dose group supporting the notion that at least at this dose part of the effects are mediated by quenching NFκB activation.

An obvious question is whether the observed effects on the inflammatory mediators are clinically relevant. Reductions in aortic wall IL-6 levels may provide a mechanistic explanation for the reported reductions in circulating CRP levels during statin therapy. IL-6 is the primary determinant of the acute phase reaction, and one could speculate that quenching of IL-6 expression in the vessel wall and/or in the liver may result in reduced CRP production by the liver [21].

As yet, it is unclear whether the reductions in IL-6 levels during statin therapy are relevant for the atherosclerotic process or for the process of aneurysm progression. IL-6 is abundantly expressed in AAA, and the aneurysm has been identified as a direct source of circulating IL-6 [22]. Yet, to the best of our knowledge there is no experimental evidence in support or denial of direct involvement of IL-6 in the process of aneurysmal formation or growth.

Similarly, although consistent, albeit moderate epidemiologic associations are found between plasma IL-6 levels, and atherosclerotic disease [23], it remains to be established whether these observations reflect a causative relationship or that that they reflect co-variation of two factors. In this respect evidence from rodent studies is not helpful with separate reports suggesting respectively positive or negative roles for IL-6, and other studies failing to identify a substantial role for IL-6 at all in the atherosclerotic process [24].

The relevance of the clear reduction in aortic wall MCP-1 (CCL2) levels is equally unclear. We measured aortic wall ICAM-1 as an up-stream ligand of MCP-1 signalling [25] and found a clear reduction in the aortic wall ICAM-1 levels, consistent with reduced MCP-1 signalling. Statin therapy did not influence aortic wall macrophage content. This suggests that the reduction in MCP-1 levels is insufficient to significantly affect monocyte influx or macrophage survival, or alternatively that MCP-1 is not the primary determinant of the aortic wall macrophage content in advanced AAA disease. Support for the latter comes from experimental observations in atherosclerosis models showing that deletion of MCP-1 prevents early atherosclerotic lesion formation, but that it only minimally influences macrophage retention and activation in more advanced lesions [26].

Statin therapy did not influence the components of the adaptive immune response, yet clear effects were seen for the M1-M2 polarization with a clear shift towards M2 phenotypes [27], an effect that is generally considered atheroprotective [28] as well as beneficial in the context of tissue regeneration [29].

Changes in the macrophage polarization are paralleled by clear, but selective changes for markers such as cathepsin K and S, and ALOX5 which have been linked to macrophage differentiation [30], [31], [32] as well as aneurysmal disease. The apparent effect on cathepsin K and S but not on MMPs [33] is intriguing and may explain the reported beneficial effect of statin therapy on aneurysm progression as well as the apparent beneficial effects of statins in acute coronary syndromes [20]. The cysteine proteases cathepsin K and S are powerful collagenolytic enzymes which have been associated with plaque instability and rupture [34]. Similar observations have been made in experimental AAA models [35], [36], [37] as well as the human AAA, in which the sharp increase in aortic wall CTX collagen fragments in this disease suggests a critical role for cysteine proteases in the excess collagen turn-over that is a hallmark of the disease [12].

Expression and activity of 5-lipoxygenase in a subpopulation of macrophages has been associated with atherosclerosis progression [38] and aneurysmal disease. [31] 5-ALOX is the key enzyme in leukotriene biosynthesis and catalyzes initial steps in the conversion of arachidonic acid to these biologically active lipid mediators. This in turn has been linked to enhanced inflammation (MIP-1α) and increased MMP activity. We observed a clear reduction in ALOX-5 mRNA expression, further adding to the selective effect of statin therapy on macrophage (subclass) activation. However, this reduction is not followed by a reduction in MMP and MIP-1α levels, indicating that the reduction is either insufficient to result in down stream effects; or alternatively that ALOX-5 is not a critical determinant of MMP and MIP-1α expression in the context of human AAA.

Differences were also found with respect to the mRNA expression of Th1 specific transcription factor T-bet and aortic wall IL-13 protein levels. These observations are counter intuitive [39]. Reduced T-bet expression suggests skewing of the immune response towards a Th2 type response, yet this is not supported by the expression levels of other Th1 and Th2 markers, or by the reduction in IL13 protein levels. Evidence from animal studies characterizes IL-13 as an anti-inflammatory Th2 type cytokine that reduces macrophage activation, thus suggesting a less prominent Th2 response. These observation for T-bet and IL-13 may suggest that these latter pathways are overruled by more dominant inflammatory pathways.

An obviously critical point is whether these observations for the aneurysm wall are of clinical benefit. Statins have been shown to reduce aneurysm *formation* in established models of the disease [36], [40]. The effect of statins on AAA *progression* in humans on the other hand, is controversial. A meta analysis of four published small observational studies indicates clear evidence for a beneficial effect [41]. At the same time, a large observational study from 5 centers failed to show any benefit of statin therapy on aneurysm growth [42]. As such the claim that statins reduce aneurysm progression is questionable [43]. To that end we evaluated the influence of statin therapy on aneurysm progression in the control arm of the PHAST study. In this study we performed a meticulous follow up of patients under surveillance for an aneurysm. Results from this analysis paralleled those of Ferguson et al, and fail to show a beneficial effect of statin treatment on aneurysm growth.

Limitations of the study: this is an observational study and patients were not randomized to statin treatment. Decisions for statin therapy were made by the attending physicians on basis of patients characteristics as such patients on statin therapy are more likely to have a history of cardiovascular disease and/or an unfavourable cardiovascular risk profile. A second limitation of the study is that it is performed on aortic wall samples from patients undergoing elective AAA repair. Although it has been pointed out that AAA is not a primarily lipid driven disease, moderate associations exist between plasma lipids and incident AAA. Plasma total and LDL-cholesterol levels were approximately 0.5 mmol/L lower in the 20 mg group than in the control and 40 mg group, yet with the exception of a weak relationship between plasma cholesterol and aneurysm wall IL-6 levels, no relation was found between plasma lipid levels and the aortic wall parameters. Hence, although we can not fully exclude that the observed changes in part relate to changes in lipid profile or to an effect on the atherosclerotic lesions present within aneurysm we consider such effects unlikely. Another limitation of the study is that the 40 mg dose was the highest dose statin used in this study. Preclinical studies show that the threshold for an anti-inflammatory effect exceeds that for a lipid lowering effect and we can not exclude that more pronounced effects on the inflammatory cascades (and effector pathways) occur in intense dose statin regimens.

Conclusions: this observational study provides clear evidence for a dose-dependent but highly selective effect of statin therapy on vascular inflammation (“pleiotropy”) that results in a clear shift in macrophage polarization. A critical point is whether; pleiotropy is clinically relevant or merely reflects “vessel wall cosmetics”. Our findings, and those from Ferguson et al. [42] suggest that the latter is at least the case in the context of AAA progression.

## Supporting Information

Figure S1 **Statin therapy does not influence aneurysm wall leukocyte content.** Semi-quantitative analysis aortic wall T-cell (CD3), T-helper cell (CD4) and cytotoxic T-cells (CD8), B-cell (CD20), plasma cell (CD138), macrophage (CD68), neutrophil (MPO) and mast cell (tryptase) content did not indicate an effect of statin therapy on any of these markers. Non-treated controls (white bars); 20 mg statin treated (grey bars) and 40 mg statin treated (black bars).
(TIF)

Figure S2 **Representative histological images for M1 double staining (CD68/iNOS), M2 (CD68/CD163) and activated macrophages (CD68/HLA-Dr). Arrows indicate double positive cells.**
(TIF)

Figure S3 **Original Western blots for cathepsin K and S, and α-actin.** Of note quantification was performed on a luminescent image workstation, and not on the photo's.
(TIF)

Table S1 **Patient characteristics of 142 control patients under surveillance for a medium size aneurysm. Data presented as mean values ± standard deviation.**
(DOC)
