# Supplementary figures and images for: A Clinical Evaluation of Statin Pleiotropy: Statins Selectively and Dose-Dependently Reduce Vascular Inflammation
Source: PLoS One. 2013 Jan 22;8(1):e53882. doi: 10.1371/journal.pone.0053882 (PMC3551939; doi:10.1371/journal.pone.0053882)

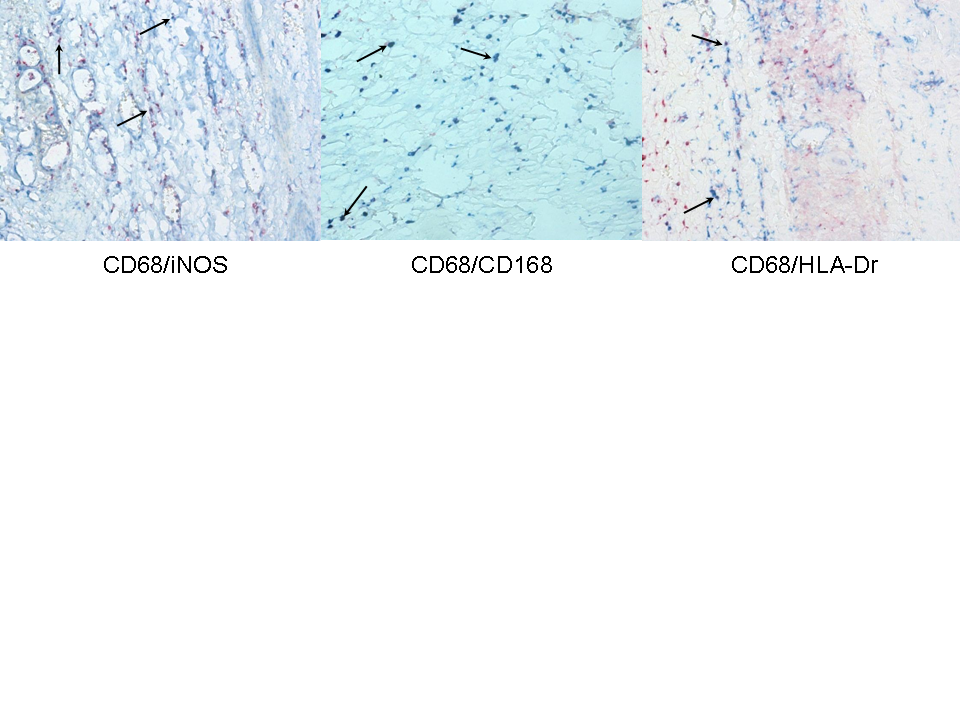

Supplement: Figure S2 — Representative histological images for M1 double staining (CD68/iNOS), M2 (CD68/CD163) and activated macrophages (CD68/HLA-Dr). Arrows indicate double positive cells. (TIF) [file pone.0053882.s002.tif]
