# Supplementary material for: A Clinical Evaluation of Statin Pleiotropy: Statins Selectively and Dose-Dependently Reduce Vascular Inflammation
Source: PLoS One. 2013 Jan 22;8(1):e53882. doi: 10.1371/journal.pone.0053882 (PMC3551939; doi:10.1371/journal.pone.0053882)
Supplement: Supplementary file 2 [file pone.0053882.s004.doc]

Supplemental Table 1. Patient characteristics of 142 control patients under surveillance for a medium size aneurysm. Data presented as mean values ± standard deviation.

	Statin treatment		
	**yes** n=103	**no** n=39	*p-value*
Age (years)	71 (± 7)	69 (± 8)	*0.359*
Male gender	89%	83%	*0.283*
Baseline diameter (mm)	43.4 (± 5.5)	42.2 (± 5.5)	*0.265*
BMI (kg/m2)	27 (± 4)	26 (± 4)	*0.791*
Smoking			*0.058*
current	31%	51%	
ever	55%	44%	
never	14%	5%	
Pack years	40 (± 26)	38 (± 26)	*0.721*
Blood pressure			
Systolic	149 (± 24)	146 (± 18)	*0.593*
Diastolic	85 (± 11)	84 (± 7)	*0.521*
Diabetes Mellitus	15%	13%	*0.710*
COPD	47%	41%	*0.589*
History of cardiovascular disease	55%	32%	***0.014***
Family history of AAA	26%	31%	*0.692*
Antihypertensives	76%	51%	***0.005***
Cholesterol (mmol/L)	4.5 (± 1.2)	5.7 (± 1.1)	***<0.001***
ApoA-1 (mmol/L)	1.4 (± 0.3)	1.5 (± 0.2)	***0.033***
ApoB (mmol/L)	0.9 (± 0.3)	1.1 (± 0.3)	***<0.001***
Triglycerides (mmol/L)	2.3 (± 2.4)	2.0 (± 0.7)	*0.516*
Creatinine (ml/min)	95 (± 22)	92 (± 27)	*0.523*
